# The relationship between intimate partner violence and alexithymia in women of reproductive age and affecting variables: A cross-sectional study

**DOI:** 10.1007/s11845-026-04356-5

**Published:** 2026-04-06

**Authors:** Fatma Başaran

**Affiliations:** Faculty of Health Sciences, Department of Midwifery, Ağrı İbrahim Çeçen University, Ağrı, Türkiye

**Keywords:** Alexithymia, Intimate partner violence, Türkiye, Women

## Abstract

**Aim:**

Studies have shown that intimate partner violence has negative effects on women’s health. Understanding alexithymia and associated factors with depressive symptoms as a result of intimate partner violence may help develop screening strategies to identify at-risk women. The aim of this study was to examine the relationship between intimate partner violence and alexithymia in women of reproductive age. In addition, the study aimed to identify the variables affecting this relationship.

**Methods:**

This cross-sectional study was conducted with 1493 married and literate women aged 15–49 years. "Introductory Information Form", "HITS Domestic Violence Screening Tool", and "The Toronto Alexithymia Scale (TAS-20)" were used to collect the study data.

**Results:**

The women had a mean score of 5.60 ± 2.59 on the HITS Domestic Violence Screening Tool and were determined to have low levels of violence, while they had a mean score of 52.51 ± 9.45 on The Toronto Alexithymia Scale and had moderate alexithymia. As the domestic violence levels of women increased, it was determined that they had difficulty in recognizing and verbalizing emotions, had externally oriented thinking styles, and accordingly their alexithymia levels increased. Age, type of marriage, years of marriage, occupation, educational status, spouse’s educational status, and income status were determined to be effective variables on intimate partner violence and alexithymia.

**Conclusions:**

Intimate partner violence is associated with higher alexithymia levels, and awareness-based interventions are recommended to reduce exposure to violence, which has many negative effects on women’s health.

## Introduction

Violence against women, particularly intimate partner violence (IPV), is a major public health problem worldwide and a violation of women’s human rights. Worldwide, almost one-third (27%) of women aged 15–49 in a relationship have experienced some form of physical and/or sexual violence by an intimate partner [1]. This violence occurs as a result of physically, sexually, and psychologically harmful behaviors, as well as emotional and economic abuse and controlling behaviors as a result of marriage, cohabitation, or any other form of union [2]. The COVID-19 pandemic has exacerbated the already heavy burden of violence, which has many short and long-term negative effects on the physical and mental health of women and children worldwide [3]. In a study conducted by Shitu et al. (2021) during the COVID-19 pandemic, it was determined that 18.2% of women experienced physical violence, 36% of emotional violence, and 29.6% of sexual violence in the last 12 months, while 12.9% experienced all three forms of violence in this process. In a previous study, it was determined that the emergence of the COVID-19 pandemic had a significant impact on violence and was an independent predictor of violence [4].

SDG-5 goal of the Sustainable Development Goals (SDGs), which is a global strategy, aims to eliminate all forms of violence against women and girls by 2030. This strategy with the indicator "SDG-5.2.1: Proportion of ever-partnered women and girls aged 15 years and older subjected to physical, sexual or psychological violence by a current or former intimate partner in the previous 12 months, by the form of violence and by age", focuses specifically on intimate partner violence and requires countries to report regularly [5]. A review of the literature reveals that intimate partner violence has many short and long-term negative effects on the physical, mental, sexual, and reproductive health of women and girls, including injuries, depression, stress and anxiety disorders, unwanted pregnancy, unwanted abortion, gynecological problems, sexually transmitted infections, sleep difficulties, eating disorders, homicide, or suicide [2, 6, 7]. In a study in which 35 cohort studies were analyzed to determine the negative health consequences of intimate partner violence, it was determined that there was a positive and statistically significant relationship between intimate partner violence and depressive symptoms [6]. In a study conducted by Signorelli et al. (2020) with 57 victims of intimate partner violence and 57 controls groups, it was determined that there were significant differences between the two groups in terms of depression, posttraumatic stress disorder, and alexithymia, and alexithymia played a role in the development of posttraumatic stress disorder in the abused women group [8].

Alexithymia, which is defined as the inability to understand, process, or identify emotions, is recognized as a risk factor for medical or psychiatric disorders [9]. It was stated that there is a link between intimate partner violence, which brings along depressive symptoms, and alexithymia moreover, alexithymia is not only considered a risk factor for intimate partner violence but can also occur as a consequence of violence and trauma [10]. Dubé et al. (2023) stated that alexithymia significantly mediated the relationship between intimate partner violence and posttraumatic stress disorder symptoms [11]. The reproductive age, which covers a long and vital period in a woman’s life, brings along many relationships and partnerships. Along with the roles such as adolescence, marriage, pregnancy, motherhood, etc., because of the introduction of intimate partner violence into women’s lives, many negative consequences emerge, primarily the physical and mental health of women. When the literature was reviewed, no study was found that evaluates the relationship between intimate partner violence and alexithymia in women of reproductive age, and the variables affecting this relationship. Therefore, this study aims to fill the research gap in the literature by evaluating the relationship between intimate partner violence and alexithymia in women of reproductive age and the variables affecting it. For this reason, efforts have been made to seek answers to the following questions:

- What is the level of intimate partner violence among women of reproductive age?
- What is the level of alexithymia in women of reproductive age?
- What is the relationship between intimate partner violence and alexithymia in women of reproductive age?
- What are the variables affecting intimate partner violence and alexithymia levels of women of reproductive age?

## Materials and methods

### Study design

This cross-sectional research was conducted between May 2023 and July 2023 to examine the relationship between intimate partner violence and alexithymia in women aged 15–49, and the variables affecting this relationship.

### Sampling and participants

The population of the study consisted of 20,360,396 individuals who were married in Türkiye in 2021, according to Address Based Population Registration Statistics [12]. The sample of the study consists of 1504 people with a confidence interval of 98% and a margin of error of 3%. While those between the ages of 15–49, female gender, married, and literate were included in the study, those who were single at the time of the study were excluded. Participants were included in the research online, and the research was terminated when 1504 participants were reached. Incomplete or incorrect data (*n* = 7) and surveys belonging to unmarried women (*n* = 4) were excluded from the data set before the analyses. The research was conducted with a total of 1493 women.

### Data collection tools

"Introductory Information Form", "HITS Domestic Violence Screening Tool (HITS)" and "The Toronto Alexithymia Scale (TAS-20)" were used to collect the study data.

### Introductory information form

This form consists of a total of 9 questions inquiring about women’s sociodemographic characteristics such as age, type of marriage, duration of marriage, educational status, and occupation.

### HITS Domestic Violence Screening Tool (HITS)

The Turkish adaptation of the scale developed by Sherin et al. (1998) was conducted by Atabay et al. in 2021. The scale is graded on a 5-point Likert scale and consists of a total of 4 items. The total score is obtained by summing the scores obtained from each item in the scale. Each item is scored between 1–5, and a score between 4–20 is obtained from the scale. A high score is considered a high risk for violence. Cronbach’s Alpha value of the scale is 0.88 [13]. In this study, Cronbach’s Alpha value was determined as 0.90.

### The Toronto Alexithymia Scale (TAS-20)

Developed by Bagby et al. (1994) [14], the Turkish adaptation of the scale was conducted by Güleç et al. in 2009. The scale consists of a total of 20 items and is graded on a 5-point Likert scale. The scale consists of three subscales: “Difficulty Identifying Feelings (1, 3, 6, 7, 9, 13, 14)”, “Difficulty Describing Feelings (2, 4, 11, 12, 17)”, and "Externally Oriented Thinking (5, 8, 10, 15, 16, 18, 19, 20)". Some items of the scale (4, 5, 10, 18, and 19) are scored in reverse order. The highest score that can be obtained from the scale is 100, and the lowest score is 20. High scores indicate a high level of alexithymia. Cronbach’s Alpha value for the overall scale is 0.78; for the subscales, it is 0.80, 0.57, and 0.63, respectively [15]. In this study, Cronbach’s Alpha values were 0.76 for the overall scale, 0.86, 0.57, and 0.79 for the sub-scales.

### Statistical analysis

The data obtained from the research were transferred to the computer environment and evaluated by IBM SPSS (version 25.0) statistical package program. Normality tests were performed in the analysis of the data, and it was determined that the data showed normal distribution. One-way ANOVA was used in the presence of three or more independent variables, an Independent Sample t-test was used in the presence of two independent variables, and a Pearson Correlation test was used to evaluate the relationships between scales and subscales. The statistical significance level was accepted as p < 0.05.

### Ethical considerations

Written permission was obtained from the scale owners via e-mail before the study. Ethics committee approval was obtained from the Scientific Research Ethics Committee of the university where the study was conducted (Date: 25.05.2023, Number: 129). Consent was obtained from all women through an online survey.

### Data collection

After obtaining ethics committee permission, the data were collected by the researcher through a Google form prepared in the form of a web survey that was open to everyone within the scope of the study. The link to this form was shared by the researcher on Facebook, Instagram, and WhatsApp accounts, and married women living in Türkiye were invited to the study.

## Results

A total of 1493 women participated in the study. The mean age of the women was 30.55 ± 7.24 years (min:17.00-max:49.00), and the mean years of marriage was 8.50 ± 7.53 years (min:1.00-max:36.00). 32.8% of women (*n* = 490) had an associate degree or higher education level, and 31.9% (*n* = 476) had a profession. It was determined that 41.5% (*n* = 620) of the husbands had an associate degree or higher education level.

The women who participated in the study received a mean score of 5.60 ± 2.59 from the HITS Domestic Violence Screening Tool and were determined to have low levels of domestic violence. In the alexithymia scale, the mean score was 52.51 ± 9.45 and it was determined that the women had moderate levels of alexithymia (Table 1).

**Table 1:** Distribution of participants’ scores on the HITS and TAS-20 Scale and its subscales

	Mean ± SD	(Min.- Max.)
HITS	5.60 ± 2.59	4.00–20.00
TAS-20	52.51 ± 9.45	24.00–88.00
Difficulty Identifying Feelings	16.51 ± 5.45	7.00–35.00
Difficulty Describing Feelings	12.80 ± 3.30	5.00–25.00
Externally Oriented Thinking	23.19 ± 3.03	9.00–35.00

It was determined that as the level of domestic violence increased, women experienced difficulties in recognizing and verbalizing emotions, had externally oriented thinking styles, and consequently their levels of alexithymia increased (Table 2).

**Table 2:** Pearson correlation coefficients between the HITS and TAS-20 Scale and its subscales (n:1493)

	HITS	TAS-20	Difficulty Identifying Feelings	Difficulty Describing Feelings	Externally Oriented Thinking
	R	r	r	r	r
HITS	1	-	-	-	-
TAS-20	0.286^**^	1	-	-	-
Difficulty recognizing emotions	0.279^**^	0.908^**^	1	-	-
Difficulty verbalizing emotions	0.245^**^	0.861^**^	0.737^**^	1	-
Externally Oriented thinking	0.122^**^	0.549^**^	0.231^**^	0.272^**^	1

*p < 0.05

It was determined that the levels of domestic violence were higher in women aged 40–49 years, those who had arranged marriages, those who had been married for ten years or more, those who had no occupation, those who had a low educational level of themselves and their spouses, and those who evaluated their income as less than their expenses. It was observed that the alexithymia levels of women aged 40–49, those who were married willingly and in any way other than arranged marriages, those who had been married for ten years or more, those who had no occupation, those with lower educational levels (both themselves and their spouses), those who evaluated their income as less than their expenses, and those who resided in the Southeastern Anatolia Region had higher levels of alexithymia (p < 0.05 for each; Table 3).

**Table 3:** Distribution of participants’ scores from HITS and TAS-20 Scale according to some sociodemographic characteristics

Variables	n (%)	HITS		TAS-20	
		Mean ± SD	t/F; p	Mean ± SD	t/F; p
Age					
15–29 (1)	786	5.20 ± 2.17	24.504;0.000*	51.85 ± 9.12	8.790;0.000*
30–39 (2)	485	5.85 ± 2.85		52.52 ± 9.73	
40–49 (3)	222	6.46 ± 3.07		54.85 ± 9.66	
^a^In-group comparison		(3–1), (3–2), (2–1)		(3–1), (3–2)	
Form of marriage					
Willingly (1)	796	4.99 ± 2.07	49.597;0.000*	51.41 ± 9.51	12.597;0.000*
Arranged marriage (2)	630	6.30 ± 2.89		53.63 ± 9.14	
Other (3)	67	6.28 ± 3.40		55.15 ± 10.17	
^a^In-group comparison		(2–1), (1–3)		(2–1), (1–3)	
Year of marriage					
1–3 years (1)	532	4.97 ± 1.90		51.21 ± 9.11	
4–9 years (2)	436	5.48 ± 2.58	39.78;0.000*	52.69 ± 9.68	9.38;0.000*
10 years and above (3)	525	6.35 ± 3.00		53.70 ± 9.47	
^a^In-group comparison		(2–1), (3–1), (3–2)		(2–1), (3–1)	
Profession					
Employed	476	5.00 ± 2.19	65.266;0.000*	50.35 ± 10.03	10.06;0.002*
Unemployed	1017	5.88 ± 2.72		53.53 ± 9.00	
Education status					
Literate (1)	166	6.53 ± 3.09		55.29 ± 7.75	
Primary School (2)	229	6.53 ± 3.10		54.36 ± 9.32	
Middle School (3)	270	6.03 ± 2.77	22.567;0.000*	53.49 ± 9.39	12.153;0.000*
High School (4)	338	5.24 ± 2.02		52.65 ± 8.16	
Bachelor’s degree (5)	467	4.83 ± 1.97		50.09 ± 10.11	
Master’s degree and above (6)	23	5.52 ± 3.72		49.73 ± 14.55	
^a^In-group comparison		(1–4), (1–5), (2–4), (2–5), (3–4), (3–5)		(1–5), (2–5), (3–5), (4–5)	
Spouse’s education level					
Literate (1)	81	6.06 ± 2.94		54.22 ± 8.91	
Primary School (2)	165	7.20 ± 3.25		54.92 ± 8.38	
Middle School (3)	258	6.34 ± 2.90	30.816;0.000*	54.20 ± 9.27	11.758;0.000*
High School (4)	369	5.53 ± 2.36		53.36 ± 8.77	
Bachelor’s degree (5)	572	4.81 ± 1.98		50.62 ± 9.88	
Master’s degree and above (6)	48	5.22 ± 2.16		48.37 ± 9.57	
^a^In-group comparison		(1–5), (2–1), (2–3), (2–4), (2–5), (2–6), (3–4), (3–5), (4–5)		(1–6), (2–5), (2–6), (3–5), (3–6), (4–5), (4–6)	
Income status					
Income less than expenses (1)	369	6.00 ± 2.79		53.78 ± 9.14	
Income equals expenses (2)	832	5.49 ± 2.38	5,947;,003*	52.25 ± 9.39	4.803;0.008*
Income more than expenses (3)	292	5.40 ± 2.86		51.67 ± 9.88	
^a^In-group comparison		(1–2), (1–3)		(1–2), (1–3)	
Province of residence					
Mediterranean Region (1)	85	5.15 ± 2.11		51.22 ± 10.12	
Eastern Anatolia Region (2)	806	5.77 ± 2.61		52.40 ± 9.11	
Aegean Region (3)	51	5.43 ± 2.27		51.41 ± 7.05	
Southeastern Anatolia Region (4)	322	5.37 ± 2.70	1.911; 0.76	54.45 ± 10.17	4.642;0.000*
Central Anatolia Region(5)	101	5.81 ± 2.84		52.49 ± 9.65	
Black Sea Region (6)	31	5.58 ± 2.21		49.22 ± 9.89	
Marmara Region (7)	97	5.22 ± 2.42		49.83 ± 8.81	
^a^In-group comparison		-		(4–7)	

^a^ Scheffe test

*p < 0.05

## Discussion

The reproductive age is the period when women are most at risk of health problems and intimate partner violence. Violence, which negatively affects women’s physical, psychological, sexual, and reproductive health, deeply affects not only the victims of violence but also many individuals, especially children [16]. It was determined that the perceived domestic violence levels of the women who participated in our study were low. When the literature was examined, it was determined that the prevalence of intimate partner violence in women of reproductive age varied but was generally at a moderate level [7, 17–19]. The possible reason for this difference is thought to be due to differences in sample size, study population, socio-cultural differences between countries, and differences in the measurement tools used for intimate partner violence. Furthermore, it is thought that women are not aware of the actual extent of violence they are exposed to or are afraid to express it.

Alexithymia, which is named in various ways, such as not being able to name and express emotions, not being able to separate emotions from each other, or living without being aware of emotions, brings along many psychological symptoms in women [8]. It was determined that the women who participated in our study had a mean score of 52.51 ± 9.45 on the alexithymia scale and had moderate alexithymia. When the studies conducted with women with a history of intimate partner violence and the control group were considered, it was concluded that the mean alexithymia scale score was higher in women who were victims of violence than in the control group and was generally at a moderate level or higher. In women without a history of violence, the mean alexithymia scale score was observed to be below the medium level [8, 20, 21]. Our study findings show similarity with the results of the studies in the literature, and it is considered that the violence levels of the women who participated in our study are higher than the stated results.

Intimate partner violence is a risk factor for depressive symptoms and alexithymia [20]. In our study, it was determined that as the violence levels of women increased, they had difficulty in recognizing and verbalizing emotions, had externally oriented thinking styles, and as a result, their alexithymia levels increased. In a study conducted by Popa-Velea et al. (2020) with women victims of intimate partner violence, it was determined that there was a significant correlation between intimate partner violence and alexithymia and externally oriented thinking sub-scale, while there was a weak correlation between violence and difficulty in recognizing and verbalizing emotions sub-scale [21]. In another study, perpetrators of intimate partner violence were observed to have higher levels of alexithymia than the control group [22]. Our study findings are consistent with the results of other studies; it was observed that there is a strong association between violence and alexithymia, and those who perpetrate or are exposed to violence are at risk for alexithymia.

As well as the contextual characteristics of women and their partners, intimate partner violence occurs due to their developmental characteristics, behaviors, relationships, and interactions [23]. In our study, it was determined that violence levels were higher among women aged 40–49 years, those who had been married for ten years or more, those who had arranged marriages, those who had no occupation, those who had a low educational level of themselves and their spouses, and those who evaluated their income as less than their expenses. In a study conducted in Liberia in which intimate partner violence in the last 12 months was evaluated among women in the reproductive age group, it was determined that the level of violence was higher among women aged 40–49 years and those who had a low educational level of themselves and their spouses, whereas there was no relationship between the employment status of women and violence [17]. In a study conducted in Ethiopia, it was stated that women aged 35 and over were 2.42 times more likely to experience violence than women aged 19 and under [4]. In a cross-sectional study conducted by Kabir et al. with women of reproductive age, it was determined that there was a substantial level of significance between education level, employment status, age, education level of the partner, and intimate partner violence. It was stated that women aged 15–24 years and women from poorer and middle-class backgrounds were more likely to be exposed to intimate partner violence, and approximately 48% of women who completed secondary education were exposed to violence [18]. In another comprehensive study, it was reported that there was a significant relationship between low educational level and intimate partner violence. Low socioeconomic status and arranged marriages were found to be significant risk factors for intimate partner violence [24]. The results of previous studies show similarity with our study in general. It is considered that the sociodemographic characteristics of women and their partners are important variables in intimate partner violence. A good education and income level empower women in preventing or eliminating violence, and supporting women and girls will make a great contribution in this regard.

Alexithymia, which can be both the cause of violence and the consequence of violence, emerges as a personality trait associated with difficulties in identifying and communicating personal emotions depending on various factors [10]. In our study, it was determined that the alexithymia levels were higher in women aged 40–49 years, those who had been married for ten years or more, those who were married willingly and in any way other than arranged marriages, those who did not have any occupation, those who had low educational level themselves and their spouses, those who evaluated their income as less than their expenses and those who resided in the Southeastern Anatolia Region. In a study conducted in Iran, no significant relationship was reported between education level and alexithymia, while a significant relationship was determined between age and alexithymia [25]. In another study on perpetrators of violence, a significant relationship was stated between age and alexithymia; however, no significant relationship was observed between age, marital status, relationship duration, employment status, and alexithymia [10]. No study examining the relationship between the characteristics of women of reproductive age and alexithymia was found. It is concluded that there is a need for studies addressing the relationship between the characteristics of women and alexithymia.

## Limitations

This research was conducted online via social media platforms, which constitutes an important limitation of the study. The sample included only women who had access to the internet and were able to use digital tools such as smartphones, computers, or tablets to complete the survey. Therefore, women without access to digital technologies or social media platforms may have been underrepresented, leading to potential selection bias. In this respect, the findings may not fully represent the general population of married women in Türkiye, and the generalizability of the results should be interpreted with caution. In addition, participation was voluntary and based on self-report, which may have introduced response bias. Moreover, the majority of participants were from the Eastern and Southeastern Anatolia Regions, which may further limit the representativeness of the sample.

Despite these limitations, the study has several strengths. The relatively large sample size increases the statistical power and credibility of the findings. In addition, the use of valid and reliable measurement tools contributes to the robustness and methodological rigor of the study.

## Conclusion

In our study, it was determined that women had low levels of intimate partner violence and moderate levels of alexithymia. Age, type of marriage, years of marriage, occupation, educational status, spouse’s educational status, and income status were identified as significant variables associated with both intimate partner violence and alexithymia. Furthermore, higher levels of intimate partner violence were associated with higher levels of alexithymia in women.

Intimate partner violence is a serious public health problem that negatively affects the physical, mental, sexual, and reproductive health of millions of women worldwide. Therefore, it is important to conduct further longitudinal and experimental studies to better understand the direction of the relationship between intimate partner violence and alexithymia and to develop effective intervention strategies.

Health professionals play a crucial role in addressing intimate partner violence. In particular, midwives and nurses working in primary care settings are in a key position for routine screening of IPV, early identification of at-risk women, and timely referral to appropriate psychosocial support services. Strengthening the competencies of midwives and nurses in these areas may contribute to reducing the adverse effects of violence on women’s health and improving overall well-being.

## Data Availability

The data supporting this study’s findings are available on request from the corresponding author. The data are not publicly available due to privacy or ethical restrictions.
